# Structured implementation of the Supportive and Palliative Care Indicators Tool in general practice – A prospective interventional study with follow-up

**DOI:** 10.1186/s12904-022-01107-y

**Published:** 2022-12-01

**Authors:** Kambiz Afshar, Katharina van Baal, Birgitt Wiese, Tanja Schleef, Stephanie Stiel, Gabriele Müller-Mundt, Nils Schneider

**Affiliations:** Institute for General Practice and Palliative Care, Hannover Medical School, Carl-Neuberg-Straße 1, 30625 Hannover, Germany

**Keywords:** Palliative care, End-of-life care, General practice, Primary care, Patient-centred care

## Abstract

**Background:**

General practitioners (GPs) play a key role in the provision of primary palliative care (PC). The identification of patients who might benefit from PC and the timely initiation of patient-centred PC measures at the end of life are essential, yet challenging. Although different tools exist to support these key tasks, a structured approach is often missing.

**Objective:**

The study aimed at implementing the German version of the Supportive and Palliative Care Indicators Tool (SPICT-DE™) in general practices, following a structured and regional approach, in order to evaluate the effects of this tool on the identification of patients with potential PC needs and the initiation of patient-centred PC measures.

**Methods:**

The intervention of this mixed-methods study comprised a standardised training of 52 GPs from 34 general practices in two counties in Lower Saxony, Germany, on the use of the SPICT-DE™. The SPICT-DE™ is a clinical tool which supports the identification of patients with potential PC needs. Subsequently, over a period of 12 months, GPs applied the SPICT-DE™ in daily practice with adult patients with chronic, progressive diseases, and completed a follow-up survey 6 months after the initial patient assessment. The outcome parameters were alterations in the patient’s clinical situation, and the type and number of initiated patient-centred PC measures during the follow-up interval. Additionally, 12 months after the standardised training, GPs provided feedback on their application of the SPICT-DE™.

**Results:**

A total of 43 GPs (*n =* 15 female, median age 53 years) out of an initial sample of 52 trained GPs assessed 580 patients (*n* = 345 female, median age 84 years) with mainly cardiovascular (47%) and cancer (33%) diseases. Follow-up of 412 patients revealed that 231 (56%) experienced at least one critical incident in their disease progression (e.g. acute crisis), 151 (37%) had at least one hospital admission, and 141 (34%) died. A review of current treatment/medication (76%) and a clarification of treatment goals (53%) were the most frequently initiated patient-centred PC measures. The majority of GPs deemed the SPICT-DE™ practical (85%) and stated an intention to continue applying the tool in daily practice (66%).

**Conclusions:**

The SPICT-DE™ is a practical tool that supports the identification of patients at risk of deterioration or dying and promotes the initiation of patient-centred PC measures.

**Trial registration:**

The study was registered in the German Clinical Trials Register (N° DRKS00015108; 22/01/2019).

## Background

Worldwide, the proportion of decedents who received palliative care (PC) prior to death is estimated to lie between 53 and 90% [1–5]. It is expected that the proportion of people with PC needs will rise by approximately 25% until 2040 [6], particularly with respect to patients with non-cancer diseases, such as dementia [6]. General practitioners (GPs) play a key role in the provision of end-of-life care (EoLC) and PC within the primary care setting [7–10]. Central tasks in the provision of primary PC by GPs are: (1) the identification of patients who might benefit from a PC approach, (2) conversations about death and dying, and (3) the timely initiation of PC measures (e.g. pain and symptom management, advance care planning) [9, 11, 12]. Furthermore, GPs typically play a pivotal role in the coordination of PC and the cooperation with different health care providers involved in PC, to ensure the continuity and high quality of care [13, 14].

The majority of patients in general practice who might benefit from primary and/or specialist PC are characterised by chronic, progressive, non-cancer diseases; multimorbidity; and old age [15–18]. International research and our own preliminary work underpin the need for a structured approach to the identification of patients with potential PC needs among the large and heterogeneous group of patients with chronic, progressive disease [19–22]. Indeed, the literature and practical experience demonstrate that both the identification of patients with potential PC needs and the prognostic judgments in people with declining health are very challenging, due to uncertainty and high variability, especially with regard to patients with chronic, non-cancer conditions [20, 23–25]. Specifically, different disease trajectories (depending on the underlying disease) influence predictability and care planning [26, 27], and may impede both the identification of patients with PC needs and the timely initiation of PC measures [25, 28].

Several tools exist to support the identification of patients with potential PC needs and the initiation of PC measures. These include the Prognostic Indicator Guidance (PIG), the Necesidades Palliativas (NECPAL), the RADboud indicators for Palliative Care Needs (RADPAC) [29, 30] and the Supportive and Palliative Care Indicators Tool (SPICT™) [31].

In prior studies, we have shown that the German version of the SPICT™ (i.e. the SPICT-DE™) is accepted by GPs in Germany and that it may raise practitioners’ awareness of relevant PC situations [32]. We also showed, in a pilot study, that the application of the SPICT-DE™ supports the identification of patients at risk of deterioration or dying [33]. Participating GPs considered the SPICT-DE™ to be helpful in identifying patients who might benefit from PC and practical in daily practice [33]. However, it remains unclear whether a structured and regional implementation of the SPICT-DE™ would have the same effects on the identification of patients at risk of deterioration or dying. Furthermore, it is not known if the application of the SPICT-DE™ in general practice would promote the initiation of patient-centred PC measures, and which PC measures GPs would likely initiate following a structured identification process using the SPICT-DE™.

Thus, the present study aimed at investigating whether the structured, regional implementation of the SPICT-DE™ in general practice would:

1. support GPs in identifying patients with potential PC needs at risk of deterioration or dying; and
2. promote the initiation of patient-centred PC measures following an initial assessment.

Furthermore, it aimed at describing which types of PC measures were effectively initiated by GPs, and depicting patients’ disease trajectories 6 months after their initial assessment with the SPICT-DE™. Finally, it aimed at determining whether GPs deemed the application of the SPICT-DE™ during routine consultations practical and useful.

## Methods

### Study design

The present study comprised part of the main study “Optimal Care at the End of Life” (OPAL) [34]. The study was registered in the German Clinical Trials Register (N° DRKS00015108; 22/01/2019). OPAL was a prospective, interventional, mixed-methods study with a pre-post design that aimed at optimising the care provided by GPs for patients with chronic, progressive diseases in the last phase of their lives. The description of the intervention follows the “Template for Intervention Description and Replication” (TIDieR) [35].

### Setting

OPAL followed a regional approach and was therefore implemented in two counties in Lower Saxony, Germany. Both counties are so-called “local health regions” with a special interest in facilitating cooperation between regional health care providers [36].

### Study population and recruitment

The main study population consisted of practising GPs in both counties in Lower Saxony. In October 2018, all registered GPs (*n =* 190 GPs in *n =* 124 general practices), excluding those only treating privately insured patients, were invited to participate in the study. Requests for participation were repeated via phone, letter and fax until a response was recorded by each general practice. During the recruitment phase, brief and clear study information was sent to the general practices – if necessary, repeatedly. Recruitment ended in April 2019 and is presented in detail in van Baal et al. [37].

### SPICT-DE™

The original SPICT™ (representing a clinical and practice-oriented tool) and a supplementary guide on its use were developed by the Primary Palliative Care Research Group at the University of Edinburgh in 2010 [31]. Since then, these materials have been regularly adjusted using participatory methods. The SPICT™ is a single-page sheet that can be applied in different care settings (i.e. hospitals, care facilities, general practices) [38]. It supports the identification of patients with deteriorating health due to one or more advanced conditions or a new serious illness, who might benefit from a holistic and PC approach. Specifically, the SPICT™ comprises the following three parts:

1. general indicators of poor or deteriorating health (i.e. progressive weight loss, persistent symptoms despite optimal treatment of underlying conditions, unplanned hospital admissions, increasing health problems and need for help and support);
2. clinical indicators of one or more life-limiting conditions (e.g. cancer, respiratory disease, dementia/frailty, heart/vascular disease); and
3. recommendations for possible next steps (e.g. review of current treatment and medication, specialist assessment or referral to specialist PC if symptoms or problems are complex and difficult to manage, agreement on a current and future care plan with the patient and their family, support for family carers).

The SPICT-DE™ is the German version of the SPICT™. The systematic development, adjustment and pre-testing of the SPICT-DE^TM^ for German-speaking countries was completed in 2017 via a transnational, multiprofessional and participatory approach [32]. The latest version of the SPICT-DE™ and its associated usage guide can be downloaded free of charge and without registration from the SPICT™ website [39].

### Intervention and implementation period

In spring 2019, a member of the study team visited participating GPs and their practice teams personally to administer the intervention. The intervention consisted of standardised training, which immediately preceded the implementation period. The standardised training was developed by a group including physicians, social and health scientists and teaching experts and was based on the published user guide for the SPICT-DE™ [39] and on the findings of preliminary studies [32, 33]. Interventionists were two research associates who were included in the development of the training, received supervision by a physician and followed standardised instructions for realising the training with GPs. To ensure a common understanding of the term “palliative care”, each participant was given a definition based on the German guidelines “Palliative care for patients with incurable cancer” [7] and the definition used by the World Health Organization [40]. The standardised user training (duration: approximately 15–20 minutes) demonstrated the application of the SPICT-DE™ according to the recommendations in the SPICT-DE™ Guide 2019 [39]. GPs were then asked to apply the SPICT-DE™ in daily practice over a period of 12 months (i.e. April 2019 to March 2020). Specifically, they were instructed to use the tool with all adult patients who visited the practice or were seen in a domestic setting, regardless of their place of residence (e.g. private home, nursing home, care facility), who met the following inclusion criteria: (1) age ≥ 18 years and (2) at least one cancer or non-cancer chronic, life-limiting disease according to the SPICT-DE™. Patients who had previously been referred to specialised PC and residents of hospices were excluded. For each patient who met the inclusion criteria, GPs were asked to highlight all applicable indicators and patient-centred PC measures of the SPICT-DE™. To enable the easy monitoring of GPs’ selections, we added a check box to each SPICT-DE™ indicator and measure. We also provided a free-text box for GPs to describe any additional actions that were undertaken.

### Evaluation

#### Patient-centred PC measures

The following patient-centred PC measures are included in the SPICT-DE™ [31, 32, 38]:

1. review current treatment and medication to ensure the person receives optimal care; minimise polypharmacy
2. consider specialist assessment or referral to a specialist PC team if symptoms or problems are complex and difficult to manage
3. agree a current and future care plan with the person and their family
4. clarify the need for support of family carers
5. early initiation of advance care planning if loss of decision-making capacity is likely
6. record, communicate and coordinate the care plan

#### Sociodemographic questionnaires

To gather additional information and to evaluate GPs’ application of the SPICT-DE™ in daily practice, we designed two questionnaires. The first questionnaire focused on GPs’ sociodemographic and professional data, including those relating to their practice structure. The second questionnaire was designed to acquire further information on the patients assessed with the SPICT-DE™. This semi-structured questionnaire comprised six parts, relating to:

1. sociodemographic data (i.e. gender, age, marital status, living situation);
2. main underlying disease(s);
3. treatment and medication relating to the underlying disease(s);
4. performance figures for primary PC;
5. care dependency, long-term care and home care; and
6. the presence of an advance directive and power of attorney.

#### Follow-up

Six months after the initial application of the SPICT-DE™, GPs completed a follow-up survey to provide information on any changes to the patient’s clinical situation since the initial assessment and which PC measures, if any, had been initiated. Specifically, for each patient, GPs were asked to fill out a single-page semi-structured questionnaire comprising five questions to describe any alteration in the patient’s situation over the follow-up interval. The questions investigated:

1. any acute deterioration of health;
2. any hospitalisation (i.e. number, reason, duration);
3. any alteration in the care situation (e.g. admission to a nursing home or hospice);
4. any initiation of a patient-centred PC measure according to the SPICT-DE™; and
5. death, if applicable, including the place of death (e.g. at home, in a care facility, in hospital).

#### Feedback

After the 12-month implementation period, GPs completed a questionnaire to provide feedback on the practicability of the SPICT-DE™ in everyday practice and whether they deemed the tool helpful for the identification of patients who might benefit from PC. Furthermore, GPs indicated whether the use of the SPICT-DE™ had altered their view of PC patients, in general, and the provision of PC in their practice. Finally, GPs indicated whether they could imagine using the SPICT-DE™ in daily practice in the future. GPs were able to provide free-text answers to each question.

### Data analysis

Collected data were mainly recorded in paper form. GPs had also the opportunity to use editable PDF versions of the SPICT-DE™ as well as the different questionnaires. Quantitative data were analysed using version 26 of the Statistical Package for Social Sciences (SPSS Inc., Chicago, IL/USA) and STATA version 16.

Descriptive statistics of the quantitative data were calculated, including means and standard deviations (SD), medians and interquartile ranges (IQR), and percentages. Furthermore, a mixed-model logistic regression analysis was performed to investigate the relationship between each patient-centred PC measure (target variable) and the total number of general indicators and clinical indicators of the SPICT-DE™ (predictors). The odds ratio (OR) was outlined as a measure of association between the predictors and the target variables. GP ID was included in the model as a random effect. Significance level was set to *p =* 0.05. Missing items were not replaced.

Free text answers to the open-ended questions of the feedback questionnaire were considered for further analysis. These free text answers were inserted verbatim in Microsoft Excel prior to analysis. Two members of the study team reviewed the free text answers and developed categories to summarise the responses thematically. The analysis of the free text answers followed an inductive approach inspired by conventional content analysis [41]. The analysis process started with (re)reading the free text answers to get familiar with the data. This step was followed by going through the free text answers word by word or line by line respectively depending on their type of presentation (single comments, key words or whole sentences) to develop codes inductively. The codes were then sorted thematically depending on how the codes related to or differed from each other in order to developed overarching categories. Additionally, the free text answers were sorted into negative and positive entries according to the GPs’ statements.

### Ethical approval and data protection

The ethics committee of Hannover Medical School gave ethical approval on 16 August 2018 (N° 8038_BO_K_2018). All study procedures were performed in accordance with the Declaration of Helsinki. All participating GPs provided informed consent prior to data collection. Each GP was assigned an individual ID for the purpose of pseudonymising the GP-related data. The code list was archived separately from the data collection documents. GPs and their practice teams listed each patient assessed with the SPICT-DE™ and assigned them an individual ID. These identifying lists remained at the general practices and were inaccessible to the study team. All patient data were provided completely pseudonymised, so patient identities were never revealed to the study team. Only pseudonymised data were analysed. The present study followed the data security procedure described in the study protocol of the main OPAL study [34].

## Results

### Description of the study sample: GPs

In total, 52 GPs from 34 general practices participated in the structured training. Thereof, 43 GPs (*n =* 15 female, median age 53 years, IQR = 46–59) from 32 general practices (single practices: *n =* 22) applied the SPICT-DE™ in daily practice over a period of 12 months. GPs’ median length of professional experience as a doctor was 23 years (IQR = 16–30) and, as a GP, 16 years (IQR = 8–23). Table 1 presents further characteristics of the participating GPs and their general practices.

**Table 1:** Characteristics of participating GPs (*n =* 43) and general practices (*n =* 32)

Variable		n	%
Gender	Male	28	65.1
	Female	15	34.9
PC qualification^*^	Basic course	14	32.6
	Additional qualification (incl. basic course)	8	18.6
Activity in a PC initiative^*^ (multiple responses possible)	Hospice association	6	14.0
	Quality circle	5	11.6
	Palliative network	7	16.3
	Specialist outpatient PC team	10	23.3
	Other initiative	1	2.3
Type of general practice	Single practice	22	51.2
	Group practice	17	39.5
	Joint practice	3	7.0
	Medical care centre	1	2.3
Teaching practice for medical students	Yes	12	27.9
	No	31	72.1
Care region	Medium-size city	16	37.2
	Small town	15	34.9
	Rural community	12	27.9

*PC* Palliative care; ^*^number of participants confirming this detail.

### Description of the study sample: Patients

The participating GPs (*n =* 43) assessed 580 patients (*n =* 345 female, median age 84 years, IQR = 78–89). The most common chronic conditions were cardiovascular (47%) and cancer (33%) diseases. On average, each patient had 6.5 documented diagnoses (SD = 4.9); 469 patients had three or more diagnoses (80%). Table 2 presents further characteristics of the patients, which were initially assessed with the SPICT-DE™.

**Table 2:** Characteristics of patients initially assessed with the SPICT-DE™ (*n =* 580)

Variable			n	%
Gender (*n =* 571)	Male		226	39.6
	Female		345	60.4
Marital status (*n =* 560)	Single		42	7.5
	Married		238	42.5
	Divorced		14	2.5
	Widowed		266	47.5
Living situation (*n =* 526)	At home		329	62.5
	In a long-term care facility		197	37.5
Main underlying disease according to diagnosis group and ICD-10 code (multiple responses possible) (*n =* 579)	HIV/AIDS	B20–B24	0	0.0
	Malignant neoplasm	C00–C97	192	33.1
	Cardiovascular disease	I25, I27, I28, I31, I32, I38, I42–I52	270	46.6
	Cerebrovascular disease	I60–I64, I67–I69	107	18.4
	Renal disease	N18, N28	112	19.3
	Liver disease	K70–K77	21	3.6
	Respiratory disease	J41–J45, J47, J96, E84	97	16.7
	Neurodegenerative disease	G10, G12, G20, G23, G35, G71	32	5.5
	Dementia, Alzheimer’s, senility/frailty	F00, F01, F03, G30, R54	168	29.0
Number of diagnoses* (*n =* 580)	0–3		180	31.0
	4–6		188	32.4
	7–12		144	24.8
	≥13		68	11.7
Long-term treatment of the main underlying disease (*n =* 580)	Yes		513	88.4
	No		67	11.6
Primary PC (last quarter; *n =* 506)	Yes		98	19.4
	No		408	80.6
Care dependency according to German statutory nursing care insurance (*n =* 554)	Yes		414	74.7
	No		113	20.4
	Application under consideration		27	4.9
Home care (*n =* 540)	Yes		150	27.8
	No		390	72.2
Advance directive (*n =* 522)	Yes		235	45.0
	No		194	37.2
	Discussed but not realised		93	17.8
Power of attorney (*n =* 519)	Yes		268	30.8
	No		160	51.6
	Discussed but not realised		91	17.5

*PC* Palliative care, *ICD-10* International Statistical Classification of Diseases and Related Health Problems, 10th Revision, *SPICT-DE™* German version of the Supportive and Palliative Care Indicators Tool; *totals differ from 100% due to rounding imprecision.

### Application of the SPICT-DE™ and initial assessment

The most frequently selected SPICT-DE™ general indicators were “*Depends on others for care due to increasing physical and/or mental health problems*” (81%) and “*Performance status is poor or deteriorating, with limited reversibility*” (75%). Table 3 provides a detailed overview of the general indicators selected by GPs at the initial assessment.

**Table 3:** Frequencies of SPICT-DE™ general indicators at initial assessment (*n =* 580 patients)

General indicator		n	%
Unplanned hospital admission(s)	Yes	207	35.7
	No	373	64.3
Poor or deteriorating performance status with limited reversibility (e.g. stays in bed or a chair for more than half the day)	Yes	435	75.0
	No	145	25.0
Depends on others for care due to increasing physical and/or mental health problems	Yes	467	80.5
	No	113	19.5
Family caregiver needs (more) help and support	Yes	210	36.2
	No	370	63.8
Progressive weight loss; persistently underweight; low muscle mass	Yes	167	28.8
	No	413	71.2
Persistent symptoms despite optimal treatment of underlying condition(s)	Yes	282	48.6
	No	298	51.4
Patient (or family) asks for palliative care to reduce, stop, or not have treatment	Yes	82	14.1
	No	498	85.9

The most frequently selected SPICT-DE™ clinical indicators pertained to the “Dementia/frailty” category: “*Unable to dress, walk, or eat without help*” (43%) and “*Urinary and faecal incontinence*” (28%); followed by one in the “Neurological disease” category: “*Progressive deterioration in physical and/or cognitive function, despite optimal therapy*” (26%). The clinical indicator “*Deterioration and at risk of dying with other conditions or complications that are not reversible; any treatment available will have a poor outcome*,” pertaining to the “Other conditions” category, was selected for 33% of assessed patients. Table 4 provides a detailed overview of the clinical indicators selected by GPs at the initial assessment.

**Table 4:** Frequencies of SPICT-DE™ clinical indicators at initial assessment (*n =* 580 patients)

Clinical indicator			n	%
Cancer	Functional ability deteriorating due to progressive cancer	Yes	129	22.2
		No	451	77.8
	Too frail for cancer treatment or treatment is for symptom control	Yes	53	9.1
		No	527	90.9
Dementia/frailty	Unable to dress, walk, or eat without help	Yes	252	43.4
		No	328	56.6
	Eating and drinking less; difficulty swallowing	Yes	132	22.8
		No	448	77.2
	Urinary and faecal incontinence	Yes	164	28.3
		No	416	71.7
	Not able to communicate by speaking; little social interaction	Yes	113	19.5
		No	467	80.5
	Frequent falls; fractured femur	Yes	118	20.3
		No	462	79.7
	Recurrent febrile episodes or infections; aspiration pneumonia	Yes	33	5.7
		No	547	94.3
Neurological disease	Progressive deterioration in physical and/or cognitive function, despite optimal therapy	Yes	148	25.5
		No	432	74.5
	Speech problems, with increasing difficulty communicating and/or progressive difficulty swallowing	Yes	65	11.2
		No	515	88.8
	Recurrent aspiration pneumonia; breathlessness or respiratory failure	Yes	29	5.0
		No	551	95.0
	Persistent paralysis after stroke, with significant loss of function and ongoing disability	Yes	57	9.8
		No	523	90.2
Heart/vascular disease	Heart failure or extensive, untreatable coronary artery disease with breathlessness or chest pain at rest or on minimal effort	Yes	133	22.9
		No	447	77.1
	Severe, inoperable peripheral vascular disease	Yes	23	4.0
		No	557	96.0
Respiratory disease	Severe, chronic lung disease with breathlessness at rest or on minimal effort between exacerbations	Yes	70	12.1
		No	510	87.9
	Persistent hypoxia needing long-term oxygen therapy	Yes	34	5.9
		No	546	94.1
	Condition after respiratory failure; ventilation inauspicious	Yes	2	0.3
		No	578	99.7
Kidney disease	Chronic kidney failure with deteriorating health	Yes	72	12.4
		No	508	87.6
	Kidney failure complicating other life-limiting conditions or treatment	Yes	79	13.6
		No	501	86.4
	Stopping or not starting dialysis	Yes	9	1.6
		No	571	98.4
Liver disease	Cirrhosis with one or more complications in the past year: • diuretic resistant ascites • hepatic encephalopathy • hepatorenal syndrome • bacterial peritonitis • recurrent variceal bleeds	Yes	12	2.1
		No	568	97.9
	Liver transplant not possible	Yes	4	0.7
		No	576	99.3
Other condition	Deteriorating and at risk of dying with other conditions or complications that are not reversible; any treatment available will have a poor outcome	Yes	192	33.1
		No	388	66.9

During the initial assessment, GPs recorded all patient-centred PC measures that they deemed suitable for the patient. The most commonly recorded SPICT-DE™ measures were: “*Review current treatment and medication to ensure the person receives optimal care; minimise polypharmacy*” (70%) and “*Agree a current and future care plan with the person and their family*” (70%). Table 5 provides a detailed overview of the PC measures selected by GPs at the initial assessment and follow-up.

**Table 5:** Frequencies of SPICT-DE™ patient-centred PC measures at initial assessment and follow-up

Patient-centred PC measures according to the SPICT-DE™		Deemed suitable at initial assessment with the SPICT-DE™ (*n =* 580)		Actually initiated at follow-up (*n =* 400)*	
		n	%	n	%
Review current treatment and medication to ensure the person receives optimal care; minimise polypharmacy	Yes	408	70.3	312	78.0
	No	172	29.7	88	22.0
Consider specialist assessment or referral to specialist PC team if symptoms or problems are complex and difficult to manage	Yes	170	29.3	82	20.5
	No	410	70.7	318	79.5
Agree a current and future care plan with the person and their family	Yes	408	70.3	220	55.0
	No	172	29.7	180	45.0
Clarify the need for support of family carers	Yes	263	45.3	89	22.3
	No	317	54.7	311	77.8
Early initiation of advance care planning if loss of decision-making capacity is likely	Yes	236	40.7	88	22.0
	No	344	59.3	312	78.0
Record, communicate and coordinate the care plan	Yes	261	45.0	153	38.3
	No	319	55.0	247	61.8
Other	Yes	12	2.1	12	3.0
	No	568	97.9	388	97.0

*Missing data: *n =* 12. *PC* Palliative care

### Follow-up

Six months after the initial assessment, 42 GPs from 31 general practices assessed 465 patients through a follow-up survey. Of these patients, 53 were excluded from the analysis as they did not meet the inclusion criteria (i.e. wrong assessment period). Consequently, follow-up data for 412 patients (response rate: 71%) were analysed. GPs reported that 231 patients (56%) had experienced at least one critical incident in their disease progression during the follow-up interval. In the free-text boxes, GPs noted, for example, progress or acute decompensation of the underlying chronic condition (i.e. heart failure, chronic obstructive lung disease, dementia), or a worsening of symptoms (i.e. dyspnoea, pain). Furthermore, they described general deterioration (i.e. weight loss, physical weakness) and an increased incidence of falls. Approximately one-third of patients (*n =* 151) had been admitted to hospital at least once, for a mean duration of 5 days (SD = 10). GPs reported that the care situation had altered for 11% of patients; almost three-quarters of these patients had been admitted to a care facility (nursing home: 52%; hospice: 16%; retirement home: 9%). The main reasons for these alterations were increased deterioration of health and need for care, and the requests of patients and/or family caregivers.

Table 5 contrasts the patient-centred PC measures that were deemed suitable at the initial assessment with the SPICT-DE™ with the actually initiated PC measures in the following 6 months. The most frequently initiated patient-centred PC measures were *“Review current treatment and medication to ensure optimal care; minimise polypharmacy”* (78%), *“Agree a current and future care plan with the patient and their family”* (55%), and *“Record, communicate and coordinate the care plan”* (38%). These were also the most common PC measures GPs deemed suitable for patients in the initial assessment. Furthermore, GPs planned to *“Clarify the need for support of family carers”* (45%) and engage in the *“Early initiation of advance care planning if loss of decision-making capacity likely”* (41%) in the initial assessment. The follow-up data showed that GPs implemented these PC measures less frequently than intended (each 22%).

Approximately one-third of patients (*n =* 141) died within the 6 months follow-up interval. The majority of these patients (44%) died in a nursing home, while 27% died at home. Approximately one-fifth (21%) died in hospital and 5% died in a hospice.

### Mixed-model logistic regression

A positive association emerged between the total number of general indicators and clinical indicators and the initiation of SPICT-DE™ patient-centred PC measures. The higher the number of general indicators, the higher the likelihood that one or more patient-centred PC measures were initiated (Table 6). Similarly, the higher the number of clinical indicators, the higher the likelihood that two SPICT-DE™ patient-centred PC measures were initiated: (1) *“Review current treatment and medication to ensure optimal care; minimise polypharmacy”* (OR = 1.172; *p =* 0.019) and (2) *“Consider specialist assessment or referral to specialist PC team if symptoms or problems are complex and difficult to manage”* (OR = 1.134; *p =* 0.042) (Table 6).

**Table 6:** Results of mixed-model logistic regression analyses

Patient-centred PC measures	Number of general indicators^1^		Number of clinical indicators^1^	
	OR; CI	p	OR; CI	p
Review current treatment and medication to ensure optimal care; minimise polypharmacy	1.213; 1.015–1.451	0.034*	1.172; 1.026–1.339	0.019*
Consider specialist assessment or referral to specialist PC team if symptoms or problems are complex and difficult to manage	1.865; 1.523–2.283	< 0.001*	1.134; 1.004–1.280	0.042*
Agree a current and future care plan with the patient and their family	1.393; 1.198–1.619	< 0.001*	1.100; 0.997–1.214	0.058
Clarify the need for support of family carers	1.525; 1.302–1.786	< 0.001*	1.034; 0.938–1.141	0.501
Early initiation of advance care planning if loss of decision-making capacity likely	1.604; 1.341–1.918	< 0.001*	1.079; 0.967–1.204	0.175
Record, communicate and coordinate the care plan	1.395; 1.198–1.624	< 0.001*	1.038; 0.942–1.144	0.455

*CI* Confidence interval, *OR* Odds ratio, *PC* Palliative care; *p < 0.05; ^1^*n =* 579 patients with an initial assessment and *n =* 398 patients with a follow-up assessment were included in the mixed model.

### Feedback on the application of the SPICT-DE™

Of all GPs (*n =* 52) who underwent the structured training, 47 completed the feedback questionnaire. The majority of GPs deemed the application of the SPICT-DE™ feasible in daily practice (85%) and helpful for the identification of PC patients (81%). They particularly emphasised the easy and quick administration as well as the clear structure and good outline of the tool. Some GPs described that the SPICT-DE™ indicators helped them to translate and visualise their “gut feelings” into concrete indicators for PC provision. Other GPs indicated that they would like to apply the SPICT-DE™ in combination with practice software. A few GPs suggested that the SPICT-DE™ might be especially helpful for GPs in training or young professionals. A few other GPs were critical of the SPICT-DE™, deeming it time-consuming with no added value over and above their long-standing experience in providing PC to patients at the end of life. One GP also criticised the tool for lacking a scoring mechanism. Finally, one GP indicated that he/she would use the SPICT-DE™ as the basis for billing.

Almost half of the GPs affirmed that the SPICT-DE™ altered their perception of patients with potential PC needs (47%) and their general PC provision (43%). The tool also increased their awareness of PC situations and broadened their perspective on the PC needs of patients (especially those with non-cancer diseases). In addition, GPs reported that the tool supported the communication and coordination of care, facilitating earlier referral and better cooperation with specialised PC teams. By contrast, especially GPs with long-standing experience and advanced qualifications in PC provision indicated that the tool had no effect on their perception or provision of PC. Overall, two-thirds of the GPs claimed that they could imagine applying the SPICT-DE™ in daily practice in the future.

## Discussion

This prospective, interventional, mixed-methods study aimed at investigating the effects of a structured, regional implementation of the SPICT-DE™ in general practice in Germany on (1) the identification of patients with potential PC needs and (2) the initiation of patient-centred PC measures by GPs. Additionally, it aimed at examining the disease trajectories of the assessed patients and the number and type of initiated PC measures in the 6 months following the initial assessment. Furthermore, it collected GP feedback on the application of the SPICT-DE™ in daily practice.

### Main findings

The main study findings were that the SPICT-DE™ was perceived to support: (1) the identification of patients with potential PC needs in general practice at risk of deterioration and dying and (2) the initiation of patient-centred PC measures up to 6 months following an initial assessment. A review of current treatment and/or medication and a clarification of treatment goals were the most frequently initiated patient-centred PC measures. Thus, the SPICT-DE™ was deemed practical and helpful by the majority of participating GPs.

### Identification of patients with potential PC needs

The timely identification of patients with potential PC needs may not only reduce late-life hospital admissions [42], but also improve patients’ quality of life [43]. However, the timely identification of patients with potential PC needs and making prognostic judgements about patients with different illness trajectories are often challenging for GPs, especially with regard to patients with chronic, non-cancer diseases [21, 44, 45]. Besides prognostic uncertainty, high GP workloads and limited time resources might represent additional constraints on the identification of patients with potential PC needs in general practice [46, 47].

Previous studies [32, 33, 48] have confirmed the feasibility of SPICT-DE™ application in general practice. The present study built on these prior findings by examining a structured, regional implementation of the SPICT-DE™. The results suggest that the SPICT-DE™ is practical for daily practice and helpful for the identification of PC patients at risk of deterioration and dying. This is emphasised by the high rates of critical incidents revealed in the follow-up survey. These critical incidents included acute decompensation of existing conditions, symptom worsening and increased incidence of falls, as well as numerous hospital admissions and altered care situations (most frequently, admission to a nursing home).

Based on the findings from this study, the SPICT-DE™ is not only likely to support the identification of patients with potential PC needs in the (presumably) last phase of their lives, but it may also increase GP awareness of the value of early PC integration. Participating GPs claimed that their application of the SPICT-DE™ improved their identification of patients with potential PC needs and altered their provision of PC in general practice. Such impacts may be beneficial over the long term and increase the quality of primary PC provided by GPs, even for patients who are not assessed using the SPICT-DE™.

### Initiation of patient-centred PC measures

GPs initiated specific PC measures for the majority of patients during the 6-month follow-up interval. There was only a slight discrepancy between the PC measures deemed suitable at the initial assessment with the SPICT-DE™ and the actual PC measures initiated in the 6 months to follow. In particular, participating GPs most often reviewed the treatment and/or medication plan. These results are aligned with the findings of Tetzlaff et al., showing that the most commonly initiated PC measure following SPICT-DE™ assessment was a review of the patient’s drug and non-drug therapy [48]. While polypharmacy is highly common in general practice [49], it increases the risk of side effects, lowers patients’ quality of life and may result in increased hospital admissions and mortality [50, 51]. The application of the SPICT-DE™ in general practice may contribute to increasing awareness of futile and harmful treatment and medication at the end of life.

According to the study results, the SPICT-DE™ also supported the clarification of treatment goals with patients and relatives, as well as the documentation, communication and coordination of care within advance care planning. Previous research has shown that patients who discuss their wishes for EoLC with a physician are more likely to receive care consistent with their preferences; this is often associated with less physical distress, higher quality of life and greater patient satisfaction [52, 53]. Despite growing evidence of a variety of benefits of early EoLC discussions [54, 55], such conversations are often missing or undertaken late in a patient’s disease trajectory in clinical practice [56, 57]. The SPICT-DE™ might contribute to reducing barriers to EoLC conversations in daily practice [45]. Specifically, the SPICT-DE™ Guide includes suggested approaches, words and phrases to support professionals in adequately starting such conversations with patients and their relatives [39].

Participating GPs attested to the clear structure of the SPICT-DE™, and particularly its quick and easy application. Therefore, the SPICT-DE™ could represent an effective tool for the basic assessment and documentation of patients with potential PC needs in primary care. Additionally, the tool may be used to justify the billing of initiated PC measures for patients at the end of life. Nevertheless, the structural and legal framework in Germany must be adjusted to improve the provision of primary PC [46].

### Strengths and limitations

The present study evaluated, for the first time in Germany, the effects of a structured, regional implementation of the SPICT-DE™ on the identification of patients with potential PC needs and the initiation of patient-centred PC measures. The results support the further implementation of the SPICT-DE™ in general practice. The findings relate to two counties and can therefore not be fully generalised. Furthermore, the results may have been affected by selection bias, as participating GPs may have had greater interest in PC and more advanced PC qualifications relative to the collective group of GPs in Germany. Also, the conclusions were drawn on the basis of self-report data, which may have been impacted by social desirability. Finally, the study cannot clarify in detail whether the patient-centred PC measures were effectively implemented and whether they were associated with better patient outcomes.

## Conclusions

The SPICT-DE™ is a practical and helpful tool for general practice that supports the identification of patients at risk of deterioration or dying and promotes the initiation of patient-centred PC measures. The tool seems to alter the usual identification strategy and increase GPs’ awareness of the value of PC provision. Thus, the structured implementation of the SPICT-DE™ may improve care at the end of life for patients and their relatives and optimise GPs’ provision of PC. The effect on patient-reported outcomes should be explicitly investigated in further research. Prospectively, the SPICT-DE™ should be integrated into national strategies and promoted in medical teaching and further training of physicians.

## Data Availability

The datasets generated and/or analysed during the study are available from the corresponding author upon reasonable request.

## Abbreviations

CI: Confidence interval
EoLC: End-of-life care
GP: General practitioner
IQR: Interquartile range
OPAL: Optimal Care at the End of Life
OR: Odds ratio
PC: Palliative care
SD: Standard deviation
SPICT-DE™: German version of the Supportive and Palliative Care Indicators Tool
TIDieR: Template for Intervention Description and Replication
